# PI-RADS v2.1 and PSAD for the prediction of clinically significant prostate cancer among patients with PSA levels of 4–10 ng/ml

**DOI:** 10.1038/s41598-024-57337-y

**Published:** 2024-03-19

**Authors:** Jing Wen, Wei Liu, Xiaocui Shen, Wei Hu

**Affiliations:** 1 Department of Medical Imaging, Jiangsu Vocational College of Medicine, Yancheng, China; 2 Department of Radiology, Yancheng Tinghu District People’s Hospital, Yancheng, China; 3 Department of Radiology, Yixing Traditional Chinese Medicine Hospital, Yixing, China

**Keywords:** mpMRI, Prostate cancer, PSAD, PI-RADS, Diagnostic performance, Cancer imaging, Cancer screening, Prostate cancer

## Abstract

This study intended to evaluate the diagnostic accuracy of the prostate imaging reporting and data system (PI-RADS) and prostate-specific antigen density (PSAD) for clinically significant prostate cancer (csPCa) with PSA levels of 4–10 ng/ml. Between July 2018 and June 2022, a total of 453 patients with PSA levels of 4–10 ng/ml were retrospectively included, which were randomly assigned to the training group (323 patients) and validation group (130 patients). Sensitivity, specificity, positive predictive value (PPV), and negative predictive value (NPV) with their 95% CI were calculated. The overall diagnostic performance was determined with area under the receiver operating characteristic curve (AUC), and an integrated nomogram combining PI-RADS score and PSAD was constructed and tested in a validation cohort. In the training group, the AUC for PI-RADS 2.1 and PSAD alone were 0.875 (95% CI 0.834–0.916) and 0.712 (95% CI 0.648–0.775). At the cutoff PI-RADS score ≥ 4, the sensitivity and specificity were 86.2% (95% CI 77.4–1.9%) and 84.7% (95% CI 79.6–88.8%), respectively. For PSAD, the sensitivity and specificity were 73.3% (95% CI 63.0–82.4%) and 62.1% (95% CI 55.8–68.5%) at the cutoff 0.162 ng/ml/ml. While combining PI-RADS with PSAD, the diagnostic performance was improved significantly, with AUC of 0.893 (95% CI 0.853–0.933). In the validation group, the nomogram yielded a AUC of 0.871 (95% CI 0.807–0.934), which is significantly higher than PI-RADS alone (0.829, 95% CI 0.759–0.899, *P* = 0.02). For patients with PSA levels of 4–10 ng/ml, PSAD demonstrated moderate diagnostic accuracy whereas PI-RADS showed high performance. By combination of PSAD and PI-RADS together, the diagnostic performance could be improved significantly.

## Introduction

Prostate cancer is a major health problem for men worldwide, it is estimated that 10 million living with PCa at present, and of which 7 million are living with metastatic disease^1,2^. In the management of PCa, serum prostate-specific antigen was used for screening and monitoring patients older than 50 years of age, intending to detect PCa at an early stage^3,4^. In the USA, a PSA level > 4.0 ng/ml was considered as the threshold that reference to prostate biopsy; however, PSA is not cancer-specific and elevated PSA levels can be owing to benign prostatic hyperplasia (BPH) and prostatitis^5^. Therefore, PSA demonstrates high sensitivity but low specificity, which may result in overdiagnosis and treatment of indolent prostate lesions^6^. Additionally, only 20.4% of men PSA level of 4–10 ng/ml were diagnosed with csPCa, which is called the gray zone^7^.

Over the past two decades, the application of MRI for the prostate has advanced substantially and is considered the most effective imaging modality for the detection and diagnosis of csPCa^8–10^. Furthermore, multiparametric magnetic resonance imaging (mpMRI)-based PI-RADS has been widely used in clinical practice. Nevertheless, like PSA, MRI also showed high sensitivity but lower specificity^11–13^. A recent meta-analysis including 10 studies demonstrated that the pooled sensitivity and specificity were 0.87 and 0.74 for PI-RADS v2.1, which means a number of unnecessary biopsies^14^. Moreover, mpMRI requires a long examination time of approximately 30–45 min, which is especially problematic in elderly or claustrophobic patients who cannot remain motionless for that long^15^*.* Therefore, it should balance these benefits against the potential short and long-term harms, including complications from biopsies and subsequent treatment, as well as the risk of overdiagnosis and overtreatment^16^. In recent years, PSAD has been investigated intensively and considered as an independent clinical variable or combination with others for the prediction of csPCa^17,18^. However, the diagnostic performance of PSAD and PI-RADS in patients of PSA gray zone only reported in few studies^19^. Therefore, this study aimed to assess the diagnostic performance of PI-RADS v2.1 and PSAD for the prediction of csPCa in patients with PSA levels of 4–10 ng/ml.

## Materials and methods

### Patient selection

**Figure 1:**
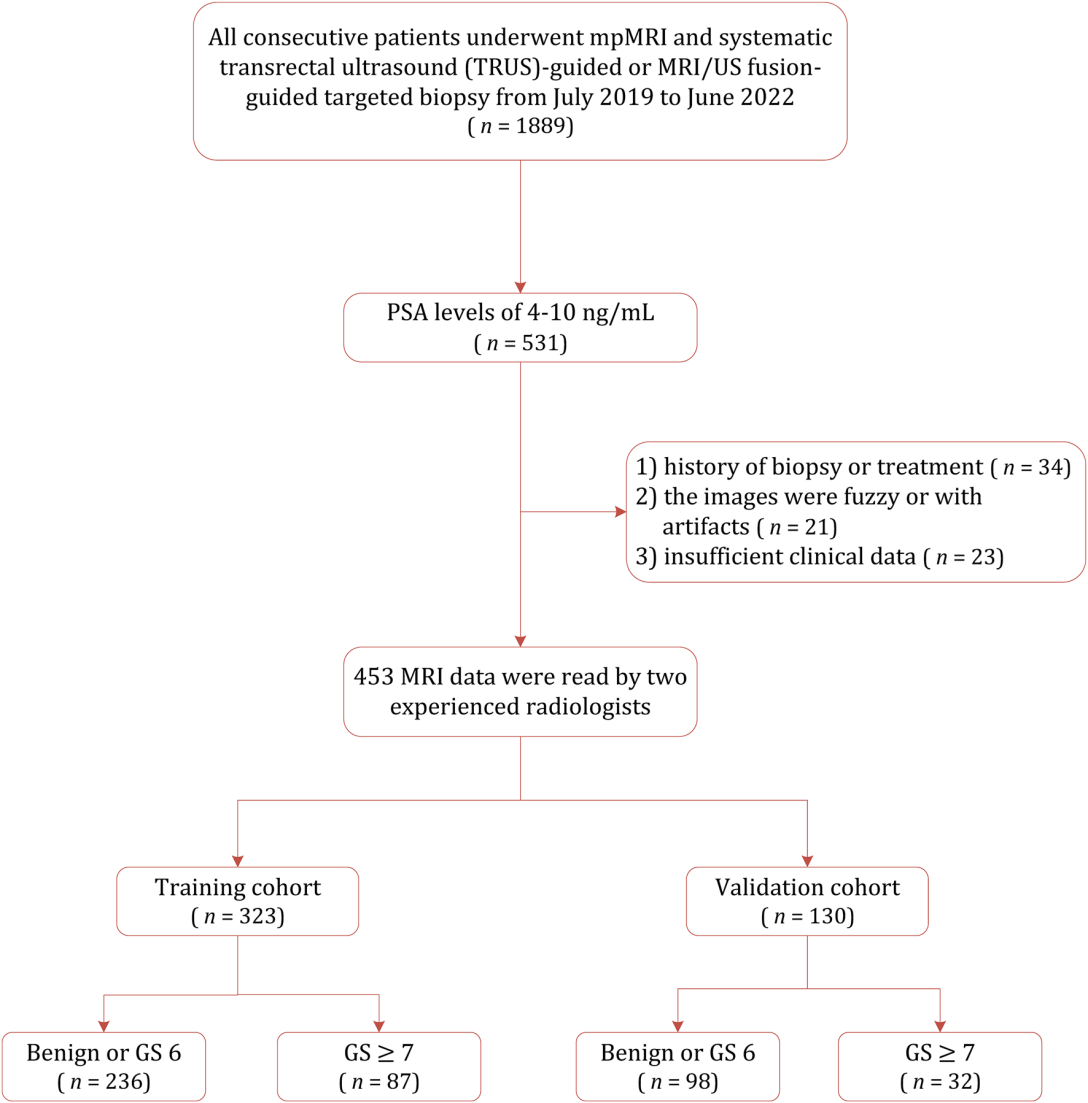
Patient selection flowchart for this study.

This study was conducted in compliance with the guiding principles of the Declaration of Helsinki, and approved by ethics committee of Jiangsu Vocational College of Medicine (JD-HG-2023-92). The requirement of written informed consent was waived by our institutional review board (Jiangsu Vocational College of Medicine) due to retrospective nature of the study. All data were collected following the Health Insurance Portability and Accountability Act (HIPAA). Between July 2018 and June 2022, 531 consecutive patients with PSA levels of 4–10 ng/ml and suspected of PCa were identified from electronic databases at our institution, all patients underwent systematic TRUS-guided biopsy after at least 4 weeks of the MRI examination. After mpMRI examination, suspicious lesions underwent MRI-transrectal ultrasonography (TRUS) fusion-guided prostate-targeted biopsy (MRGB). Of which, 78 patients were excluded for reasons as follows: (1) history of biopsy or treatment (*n* = 34); (2) the images were fuzzy or with artifacts (*n* = 21); and (3) insufficient clinical data (*n* = 23). Finally, a total of 453 patients (mean age 68.11 ± 8.81 years; median PSA 6.89, interquartile range [IQR] 5.27–8.00 ng/ml) were analyzed, of whom 323 were randomly allocated to the training group and 130 were allocated to the validation group (with a ratio of 7:3). The patient selection procedure is demonstrated in Fig. 1.

### MRI acquisition and interpretation

All patients underwent MRI examination with a 3.0 T scanner, and a pelvic 32-channel phased array coil (Ingenia 3.0 T CX Quasar Dual, Philips Medical Systems) was used. The mpMRI sequences included axial T1-weighted imaging; axial and sagittal turbo spin echo (TSE) T2-weighted imaging, single-shot echoplanar diffusion-weighted imaging, and dynamic contrast enhancement imaging. The apparent diffusion coefficient (ADC) maps were generated from DWI with ***b*** values of 0, 100, 1000, and 2000s/mm^2^. DCE was conducted immediately after injecting contrast agent of gadopentetate dimeglumine (Magnevist, Bayer Schering Pharma) with a dose of 0.1 ml/kg (2–3 ml/s), then followed by a 20 ml saline flush. Detailed MRI protocols used for imaging acquisition are provided in Table 1. MRI images were independently interpreted by two board-certified genitourinary radiologists (both with at least 5 years of experience), who were blinded to the final histopathology results and other clinical information. Each lesion was scored strictly according to the PI-RADS v2.1 criteria and assessed on a PACS workstation, and any discrepancies between radiologists were resolved through discussion.

**Table 1:** Detailed MRI sequences.

Parameter	T1WI (axial)	T2WI (axial)	T2WI (sagittal)	DWI*	DCE
Field of view (mm)	240 × 240	220 × 220	240 × 180	260 × 260	220 × 220
Acquisition matrix	276 × 406	276 × 240	104 × 125	104 × 125	124 × 121
Repetition time (ms)	566	3000	6000	6000	3
Echo time (ms)	8	100	77	77	1.45
Section thickness, no gaps (mm)	3.0	3.0	3.0	3.0	3.0
Acquisition time	1 m 10 s	4 m 6 s	3 m 42 s	3 m 54 s	6 m 7 s

*DCE* dynamic contrast enhanced imaging, *DWI* diffusion weighted imaging, *T1WI* T1-weighted imaging, *T2WI* T2-weighted imaging.

*DWI performed with ***b*** values of 0, 100, 1000, 2000s/mm^2^.

### Prostate biopsy procedure

All patients underwent standard 10-core TRUS biopsy after at least 4 weeks of the MRI examination, which was obtained in a double sextant pattern, sampling the lateral and medial portions of the apex, mid, and base of each hemi-gland. Lesions suspicious of PCa identified by MRI underwent targeted biopsy (1–2 cores/lesion, axial and/or sagittal planes). All biopsies were performed by a urologist (with 8 years of experience in prostate biopsy), utilizing an ESAOTE Mylab Twice color Doppler ultrasound device (with a 7.5-MHz transrectal end-fire probe). The location and Gleason score (GS) of an index tumor showing the highest GS were analyzed on the basis of biopsy results. When the highest GS was identified at multiple cores, the core location with the highest tumor percentage was considered the location of an index tumor. Identified lesions were annotated on the MRI T2WI by the radiologists and images of the prostate were segmented with real-time TRUS. For patients who underwent radical prostatectomy had their specimens fixed in 40% buffered formalin before being serially sectioned into horizontal slices, 3 mm thick. Subsequently, all tissues were embedded in paraffin, and sections measuring 4 μm in thickness were cut and stained with hematoxylin and eosin. An expert genitourinary pathologist (with more than 15 years of experience) evaluated the biopsy specimens and assigned each lesion a GS. Each lesion suspicious of PCa was assigned a corresponding International Society of Urological Pathology (ISUP) grade group^20^. In this study, csPCa was defined as GS ≥ 7 (3 + 4, ISUP ≥ 2), and/or extraprostatic extension (≥ pT3a). the prostate volume was calculated based on T2WI images, according to the ellipsoid volume formula: transverse width × transverse length × longitudinal height × 0.52.

### Statistical analysis

The sensitivity, specificity, positive predictive value (PPV), and negative predictive value (NPV) with their 95% CI were calculated. The optimal cutoff value for diagnostic performance was obtained by the Youden index. The overall diagnostic performance of balance between sensitivity and specificity was evaluated using the AUC, and differences were compared with DeLong’s test, with the best being defined as the largest one^21^. All analysis was performed using STATA (version 16.1), with a ***P*** value less than 0.05 indicated as statistically significant. A nomogram for the prediction of csPCa was constructed by the combination of PSAD and PI-RADS v2.1.

## Results

### Patient characteristics

Among 323 lesions in the training cohort, 105 were located in the transition zone (TZ) and 218 in the peripheral zone (PZ). At targeted biopsy, 87 were diagnosed with csPCa (26.93%) and the remaining 236 were non-csPCa (73.07%), Table 2 summarizes the demographic characteristics of included patients. There were 206 patients (45.5%) with one lesion, for the remaining 247 patients (54.5%) with multiple lesions, 181 patients had two lesions, 43 patients had three lesions, 15 patients with for lesions, and 8 patients with five lesions. Of 46 patients underwent radical prostatectomy, there were 19 categorized as GS 4 + 3, 13 categorized as 4 + 4, and 14 categorized as > 4 + 4.

**Table 2:** Characteristics of patients.

Variable	Training (*n* = 323)			Validation (*n* = 130)		
	csPCa (*n* = 87)	Non-csPCa (*n* = 236)	*P*	csPCa (*n* = 32)	Non-csPCa (*n* = 98)	*P*
Age (years, mean ± SD)	70.69 ± 8.73	67.89 ± 8.14	0.004	68.25 ± 8.79	67.84 ± 7.84	0.40
PSA (ng/mL, median [IQR])	6.74 (5.41–8.23)	6.86 (5.26–8.50)	0.63	7.13 (5.93–8.08)	6.81 (5.17–8.00)	0.26
PV (ml, median [IQR])	35.01 (26.00–67.07)	53.56 (38.71–70.38)	< 0.001	36.83 (26.50–50.98)	49.70 (36.22–70.00)	0.004
PSAD (ng/mL/mL, median [IQR])	0.18 (0.13–0.25)	0.13 (0.10–0.17)	< 0.001	0.17 (0.13–0.27)	0.13 (0.09–0.18)	0.003
Gleason score						
≤ 3 + 3	255			108		
3 + 4	26			13		
4 + 3	20			4		
4 + 4	12			1		

*csPCa* clinically significant prostate cancer, *IQR* interquartile range, *PSA* prostate-specific antigen, *PSAD* prostate-specific antigen density, *PV* prostate volume, *SD* standard deviation.

### Diagnostic performance of PI-RADS v2.1 and PSAD

At the cutoff ≥ 4, the sensitivity and specificity of PI-RADS v2.1 for PSA 4–10 ng/ml were 86.2% (95% CI 77.4–91.9%) and 84.7% (95% CI 79.6–88.8%), respectively, with PPV and NPV of 67.6% (58.0–76.1%) and 94.3% (90.3–97.0%). While using PI-RADS score ≥ 3 as the cutoff value, the sensitivity increased to 95.4% (95% CI 88.8–98.2%) but with a significant decrease in specificity (55.9%, 95% CI 49.6–62.1%). The calculated AUC for PI-RADS v2.1 was 0.875 (95% CI 0.833–0.916). While analyzed according to the anatomy zone, the calculated AUC for the TZ was 0.882 (95% CI 0.824–0.93), whereas for the PZ was 0.826 (95% CI 0.752–0.900), no significant difference was found between the two areas (P = 0.24). When used PSAD as an independent predictor for csPCa, the calculated AUC was 0.712 (95% CI 0.648–0.775) and the best cutoff value was 0.162 ng/ml/ml, at which sensitivity and specificity were 73.3% (95% CI 63.0–82.4%) and 62.1% (95% CI 55.8–68.5%). When used 0.15 ng/ml/ml as the threshold, the corresponding sensitivity and specificity were 66.7% (95% CI 55.7–76.4%) and 64.8% (95% CI 58.4–70.9%). For 46 patients underwent radical prostatectomy, 28 were scored as PI-RADS 4 and 18 were scored as PI-RADS 5, and none of these patients were categorized as ≤ 3. In these patients, 6 patients (13.0%) with PSAD ≤ 0.1 ng/ml/ml, 19 patients (41.3%) with PSAD of 0.1–0.2 ng/ml/ml, and the remaining 21 patients (45.7%) were ≥ 0.2 ng/ml/ml.

**Figure 2:**
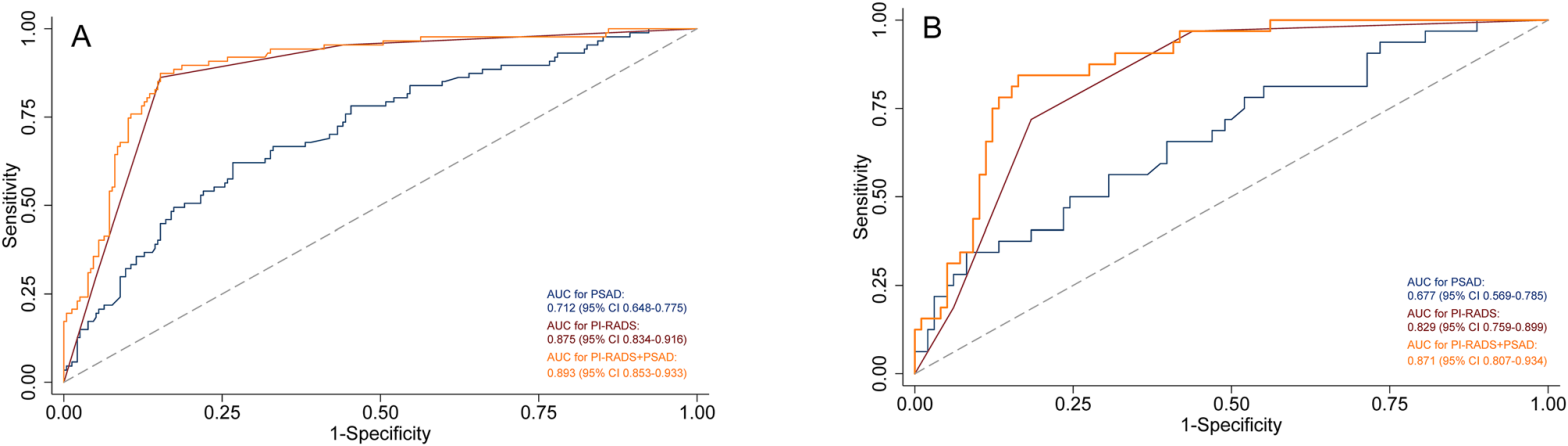
Area under the receiver operating characteristic curve for PI-RADS, PSAD, and the combination. (**A**) for the training group; (**B**) for the validation group. *PI-RADS* prostate imaging reporting and data system, *PSAD* prostate-specific antigen density.

**Figure 3:**
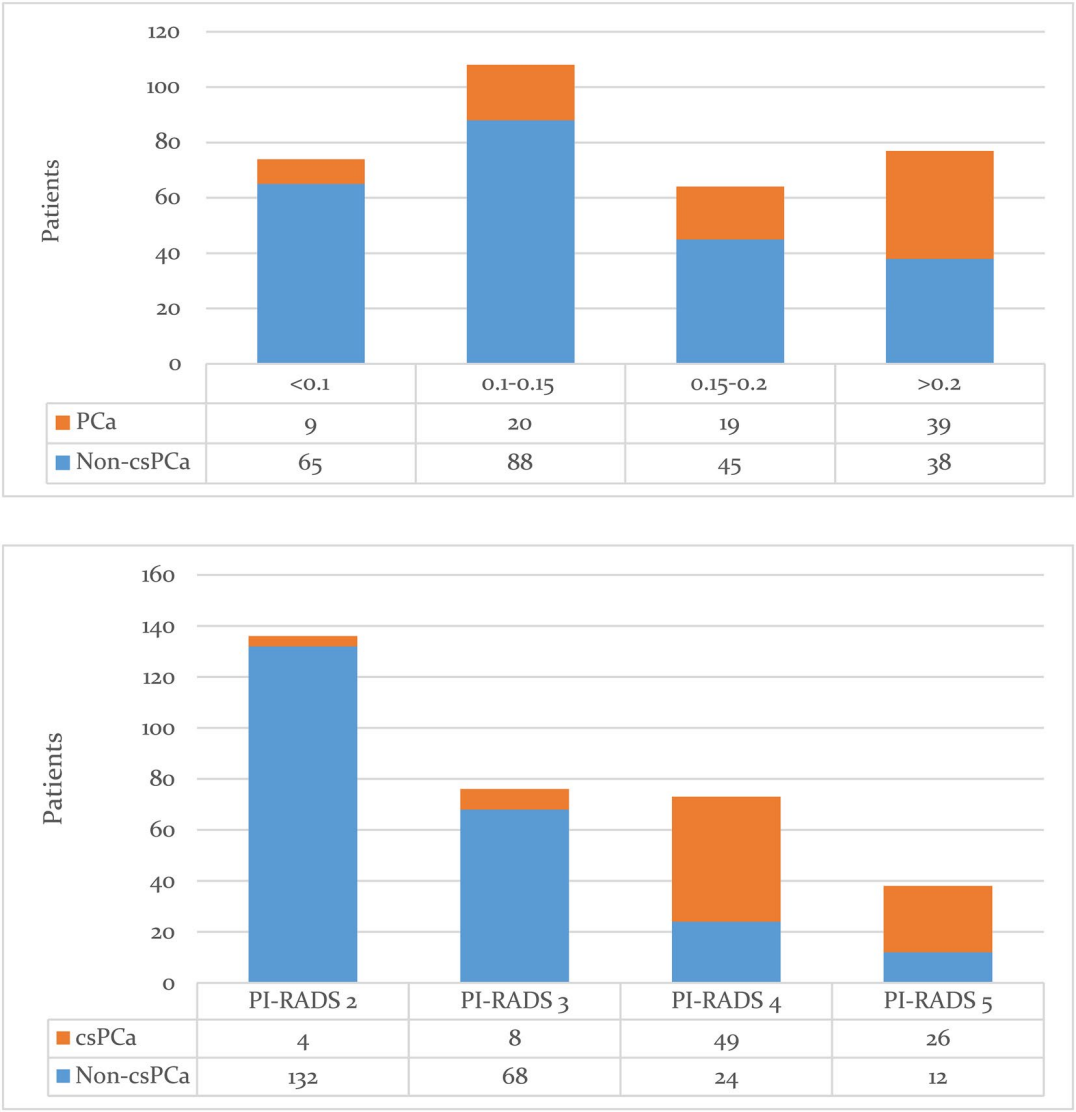
Distribution of lesions according to PI-RADS score and PSAD. *PI-RADS* prostate imaging reporting and data system, *PSAD* prostate-specific antigen density.

A nomogram was constructed by combining two covariates, and the AUC of 0.893 (95% CI 0.853–0.933) suggested a significant improvement in diagnostic performance (P = 0.01, Fig. S1). The AUCs for PSAD, PI-RADS, and combination for the training group are demonstrated in Fig. 2A. The distribution of lesions according to PI-RADS score and PSAD is presented in Fig. 3. In the validation group, the AUC of PI-RADS v2.1 was 0.829 (95% CI 0.759–0.899), which improved significantly to 0.871 (95% CI 0.807–0.934) when combined with PSAD (P = 0.02), which is presented in Fig. 2B. With combined nomogram, there were 82 csPCa were detected, whereas for PI-RADS 70 csPCa were detected. No significant difference in missing csPCa between combined nomogram and PI-RADS, with 5 vs. 6. Table 3 summarizes the detailed diagnostic performance.

**Table 3:** Diagnostic performance.

Indicator	Cutoff	Sensitivity (95% CI)	Specificity (95% CI)	PPV	NPV	AUC (95% CI)	*P* for AUC
Training cohort							
PSAD	0.162	73.3% (63.0–82.4%)	62.1% (55.8–68.5%)	41.8% (33.9–50.1%)	86.5% (80.4–91.2%)	0.712 (0.648–0.775)	P < 0.001^a^
PI-RADS	≥ 3	95.4% (88.8–98.2%)	55.9% (49.6–62.1%)	44.4% (37.1–51.8%)	97.1% (92.6–99.2%)	0.875 (0.834–0.916)	0.01^a^
	≥ 4	86.2% (77.4–91.9%)	84.7% (79.6–88.8%)	67.6% (58.0–76.1%)	94.3% (90.3–97.0%)		
PSAD + PI-RADS	–	84.7% (75.8–91.8%)	87.4% (82.4–91.3%)	71.2% (61.4–79.6%)	94.1% (90.1–96.8%)	0.893 (0.853–0.933)	–
Validation cohort							
PSAD	0.183	50.0% (31.9–68.1%)	75.5% (65.8–83.6%)	40% (24.9–56.7%)	82.2% (72.7–89.5%)	0.677 (0.569–0.785)	P < 0.001^a^
PI-RADS	≥ 3	96.9% (84.3–994%)	56.1% (46.3–65.5%)	41.9% (30.5–53.9%)	98.2% (90.4–100%)	0.829 (0.759–0.899)	0.02^a^
	≥ 4	71.9% (54.6–84.4%)	81.6% (72.9–88.1%)	56.1% (39.7–71.5%)	89.9% (81.7–95.3%)		
PSAD + PI-RADS	–	83.7% (62.7–94.7%)	84.4% (76.0–91.2%)	64.3% (48.0–78.4%)	94.3% (87.2–98.1%)	0.871 (0.807–0.934)	–

*AUC* area under the receiver operating characteristic curve, *CI* confidence interval, *NPV* negative predictive value, *REF* reference, PI-RADS prostate imaging reporting and data system, *PPV* positive predictive value, *PSAD* prostate-specific antigen density.

^a^Compared with PSAD + PI-RADS.

## Discussion

In this study, we assessed the PI-RADS v2.1 and PSAD for patients with PSA levels of 4–10 ng/ml. In the training group, our analysis based on 323 patients revealed that PI-RADS had high sensitivity and specificity for the diagnosis of csPCa at the threshold of score ≥ 4, with an AUC of 0.875. By comparison, PSAD merely yielded an AUC of 0.712, which was significantly inferior to PI-RADS (P < 0.001). When combined PI-RADS and PSAD, the AUC of 0.893 suggestted significant improvement in diagnostic accuracy as compared to PI-RADS alone (P = 0.01). We constructed an integrated nomogram on the base of PI-RADS v2.1 and PSAD and test it in the validation set. The AUC of 0.871 for validation set showed that our nomogram performed well, which was significantly superior to either PI-RADS (AUC = 0.829, P = 0.02) or PSAD (AUC = 0.677, P < 0.001). In a previous study, Han et.al. investigated mpMRI and bpMRI combined PSAD for detecting csPCa in patients with PSA serum levels of 4–10 ng/ml^19^. In their study, the AUCs for mpMRI + PSAD and bpMRI + PSAD were 0.896 and 0.907, respectively, which is consistent with our results. Nevertheless, our study included more patients; moreover, we have constructed a nomogram and tested it in the validation cohort group including 130 patients.

Screening PCa with PSA intends to detect prostate cancer at an early stage thereby reducing the disease-specific mortality. Nonetheless, low specificity of PSA is associated with substantial overdetection of harmless, low-grade cancers and side-effects related to diagnosis and treatment, especially for those patients with PSA gray zone. In this study, as many as 26.27% of patients were benign or non-csPCa, which means a number of patients may suffer unnecessary biopsies or overtreatment. The use of mpMRI as a screening tool for PCa is costly and time-consuming because of the long scanning time to acquire multiple protocols. In addition, although the PI-RADS has been released for a decade as a consensus protocol guideline, adherence varies widely among hospitals^22^. In recent years, bpMRI has been investigated intensively, and many studies reported that it has comparable performance as compared with mpMRI. However, some studies demonstrated that for lesions of PI-RADS score ≥ 3, DCE has a statistically significant ability to identify csPCa or valuable for upgrading PI-RADS score 3 to PI-RADS score 4, especially for lesions located in the PZ^23,24^. Van der Leest et al. demonstrated a “fast bpMRI” protocol that used a monoplane (axial plane) in the detection of high-grade PCa in men with a PSA value ≥ 3 ng/ml. The results showed that with fast-bpMRI, the sensitivity was not decreased compared to mpMRI (both were 95%), only with a slight decrease in specificity (65% vs. 69%). However, the results have not been widely validated^15^.

In the past several years, PSAD has been demonstrated as a promising independent predictor or combination with other clinical variables for the prediction of csPCa. In a recent meta-analysis that summarized 39 studies for risk stratification of csPCa with PSAD alone, the 3 most commonly used cutoff values for PSAD were 0.15, 0.1, and 0.2 ng/ml/ml. At cutoff values of 0.15 ng/ml/ml, the pooled sensitivity and specificity were 74% and 61%, which is comparable with our findings. However, their meta-analysis also showed widely varied optimal cutoff values and significant heterogeneity between studies. Therefore, it is unfeasible to predict csPCa only depending on PSAD alone, especially for patients with PSA gray zone or PI-RADS score 3 lesions. Our study demonstrated that by combining PSAD with PI-RADS, the diagnostic performance was improved significantly for the prediction of csPCa.

The main strength of our study was the relatively large cohort study population as compared with previous studies; moreover, we constructed a model for risk stratification of patients with PSA gray zone. Additionally, we have tested it in an independent cohort group including 130 patients. However, our study had several limitations. First, we conducted our study retrospectively and in a single center, which may result in patient selection bias and limit the generalizability of our results and conclusions. Therefore, the findings need to be validated externally. Second, using systematic and/or MRI-TRUS fusion targeted biopsy as the reference standard may miss some lesions with positive pathology that are negative in MRI. Third, the inter-reader agreement between radiologists was not investigated, and all results were from two experienced readers. Therefore, the diagnostic performance of readers with less experience for the PSA level of gray zone is unknown.

## Conclusions

The findings of the current study suggest that PI-RADS v2.1 had high diagnostic performance for csPCa with PSA gray zone. Nevertheless, the PSAD yielded only moderated accuracy as an independent predictor. By adding PSAD to PI-RADS v2.1 can significantly improve the performance for the prediction of csPCa. Further research, including prospective multicenter studies, is warranted to validate our findings.

### Supplementary Information

Supplementary Figure S1.

## Data Availability

The data that support the findings of this study are available from the corresponding author upon reasonable request.
