# Supplementary figures and images for: PI-RADS v2.1 and PSAD for the prediction of clinically significant prostate cancer among patients with PSA levels of 4–10 ng/ml
Source: Sci Rep. 2024 Mar 19;14:6570. doi: 10.1038/s41598-024-57337-y (PMC10951302; doi:10.1038/s41598-024-57337-y)

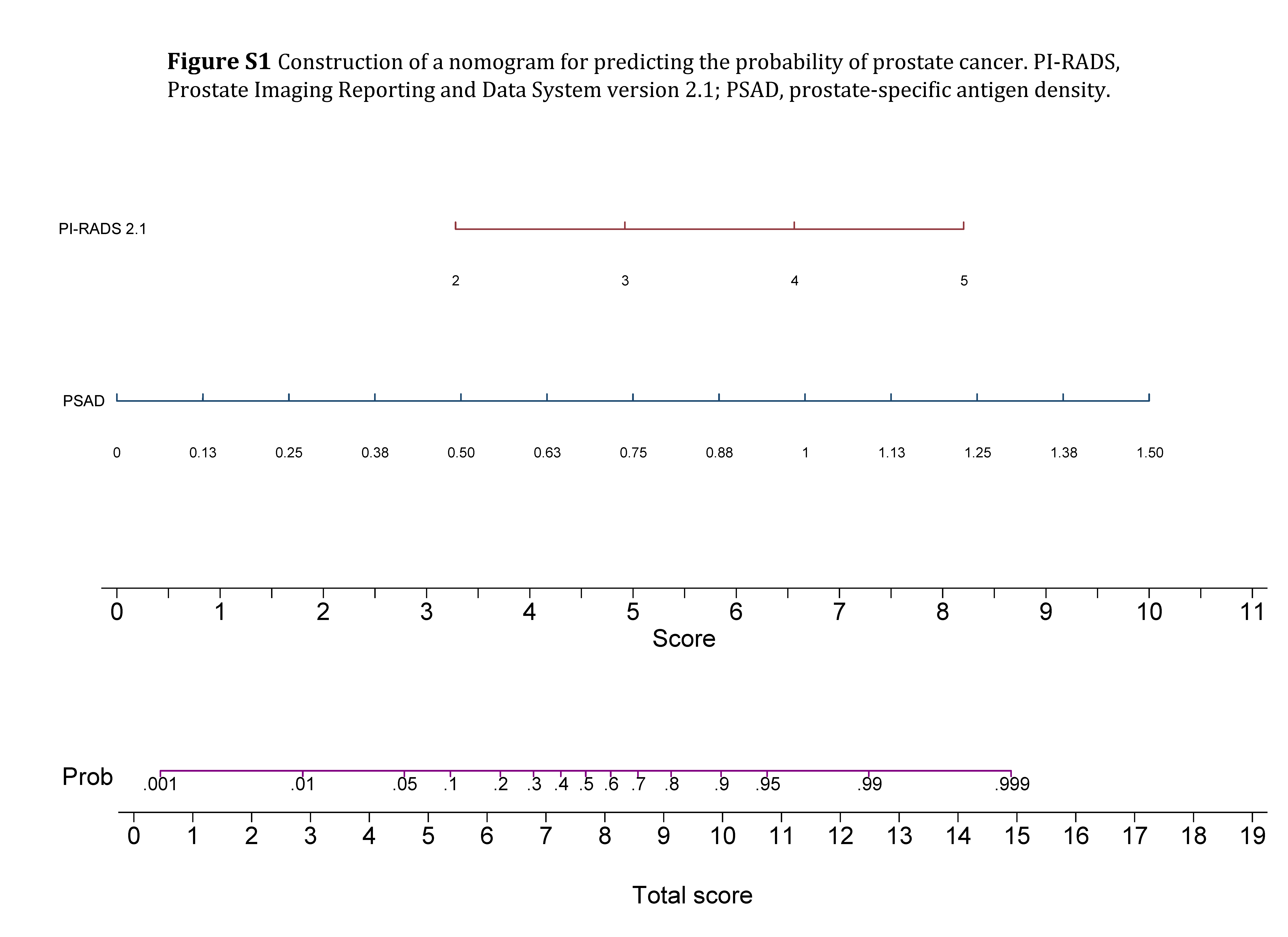

Supplement: Supplementary file 1 — Supplementary Figure S1. [file 41598_2024_57337_MOESM1_ESM.tiff]
